# Exploring adolescents' mental health in Kampala, Uganda in the context of COVID-19: a mixed methods study

**DOI:** 10.3389/frcha.2025.1419043

**Published:** 2025-02-24

**Authors:** Gideon Mbithi, Ahmed Sarki, Adam Mabrouk, Rachel Odhiambo, Mary Namuguzi, Joseph Atukwatse, Margaret Kabue, Stephen Mulupi, Amina Abubakar

**Affiliations:** ^1^ Institute for Human Development, Aga Khan University, Nairobi, Kenya; ^2^ Department of Health Studies, Royal Holloway, University of London (RHUL), Egham, United Kingdom; ^3^ School of Nursing and Midwifery, Aga Khan University, Kampala, Uganda; ^4^ Family and Youth Health Initiative (FAYOHI), Dutse, Jigawa, Nigeria; ^5^ Neurosciences Group, KEMRI-Wellcome Trust Research Programme, Centre for Geographic Medicine Research (Coast), Kilifi, Kenya; ^6^ Department of Psychiatry, University of Oxford, Oxford, United Kingdom

**Keywords:** mental health, adolescents, Uganda, COVID-19, mixed-method

## Abstract

**Background:**

Urban areas, particularly in developing nations like Uganda, face heightened risks due to poverty, unemployment, and environmental challenges, intensifying the vulnerability of urban youth to poor mental health. This study aims to determine the psychological well-being of adolescents and to assess the risks and associated factors of mental health problems among adolescents in the context of COVID-19 pandemic in Kampala City, Uganda.

**Methods:**

We conducted a cross-sectional survey of 500 adolescents aged 13–19 years residing within the five divisions of Kampala City, Uganda. We utilized standardized psychological assessment tools including the Patient Health Questionnaire, Generalized Anxiety Scale, to assess severity of depression and anxiety levels among the adolescents. A logistic regression model was used to evaluate the correlates associated with depression and generalized anxiety disorders. Variables with a *p*-value <0.25 in the univariate model were included in the multivariable regression model. Subsequently, we conducted a qualitative study using semi-structured interview guides through focused group discussions, and key informant interviews with teachers, parents, representatives of civil society organizations, and religious leaders. Qualitative data were analyzed using a thematic analysis approach.

**Results:**

The prevalence of depression was relatively higher among out-of-school adolescents at 21.5% compared to school-going adolescents at 14.0%. Furthermore, out-of-school adolescents had significantly higher anxiety scores when compared to their school-going counterparts (17.5% vs. 10.3%) respectively. Key factors identified associated with poor mental health include loneliness, being out of school, COVID-19, and familial conflicts.

**Discussion:**

This study highlights the impact of the COVID-19 pandemic on the mental well-being of adolescents in Kampala, Uganda. The reported prevalence of depression and anxiety, particularly among out-of-school adolescents, underscores the urgent need for targeted interventions in this vulnerable population. Investing in the mental well-being of Ugandan adolescents is paramount for fostering resilience and ensuring long-term success, especially in marginalized urban settings.

## Introduction

Mental health problems among adolescents have increased recently, and they are linked to significant psychosocial, economic, and physical burdens to the affected adolescents, their caregivers, and host communities (1). Across the world, mental health problems including depressive disorders, anxiety disorders and suicide, affect 10%–20% of children and adolescents (2). In sub-Saharan Africa (SSA), high prevalence estimates among different mental health problems among adolescents have been reported (3, 4). For example, a recent systematic review assessing the prevalence of mental health problems in sub-Saharan adolescents, reported a point prevalence of 26.9% for depression, and 29.8% for anxiety (5). This underscores the pressing need to address the escalating mental health challenges affecting this important demographic.

Mental health outcomes, especially in urban areas, are a cause for concern, given the evidence that populations in these regions face a high risk of poor mental health (6). Urban areas in most developing countries, including Uganda, are characterized by disproportionately high rates of poverty (7). In the specific context of Uganda, high levels of poverty in urban areas are notably linked to widespread unemployment and underemployment, creating a complex socio-economic landscape (8). This economic vulnerability, in turn, contributes to an increase in the threat of crime, and “lawlessness” within urban settings (8). The challenges in Ugandan urban areas extend beyond economic hardship, encompassing also poor housing, pollution, inadequate provisions for water, and poor sanitation (9). These social and environmental issues not only exacerbate the overall living conditions but also expose urban residents to heightened risks of poor mental health. Within this multifaceted context, the mental well-being of urban residents, particularly for the young people, emerges as a pressing concern deserving rigorous scientific attention.

The COVID-19 pandemic added additional stressors for most people particularly adolescents due to the public health measures aimed at containing the pandemic, including restriction in movements, physical and social distancing, closure of essential services, and closure of schools (10, 11). Uganda implemented one of the world's longest lockdowns, starting in March 2020 and extending until January 2022, when schools finally reopened (12). These measures put in place to curb the spread of the disease, in one way or another exacerbated the mental health problems of millions of adolescents. Additionally, factors such as fear of COVID-19 infections, loss of loved ones, and disrupted peer relationships may have contributed to the general sense of uncertainty among adolescents during the pandemic (10, 13). These events may have induced fear and set off worry and stress among some adolescents (11). Young people are highly vulnerable to events that create stress and fear (14). The prevalence of mental health problems among adolescents is noted to have increased during the pandemic (15).

The current study aimed to conduct a comprehensive mixed-method analysis to assess the status of common mental disorders among adolescents residing in urban contexts in Kampala City, Uganda. Specifically, the quantitative arm of the study aimed to assess the depressive symptoms, and anxiety symptoms of school-going adolescents compared to their out-of-school peers. Of note, it is estimated that about 19% of adolescents aged 14–17 years in Uganda are out of school (16) due to multifactorial issues, including poverty, teenage pregnancies, and being orphaned due to HIV. Examining the effect of COVID-19 pandemic on the mental health of this subgroup of adolescents provides useful data from an often overlooked group. The study also aimed to identify factors associated with mental health problems among adolescents. The qualitative arm aimed to provide a deeper understanding of the underlying factors associated with the burden of mental health problems identified in the quantitative study. By employing both quantitative and qualitative approaches, this study sought to capture both the breadth and depth of the adolescent experience in the urban contexts and in the context of the COVID-19 pandemic.

### Theoretical framework

For this study, we adapted the bio-psycho-social model which is based on the idea that an individual's general well-being is influenced by a combination of different factors (17, 18). The model illustrates the complex interplay between three primary factors as key determinants of diseases. These include biological, social, and psychological factors. The bio-psycho-social model aims to integrate the three perspectives scientifically, signaling the interconnection and interdependence of these factors.

We were interested in utilizing the bio-psycho-social model because, much like physical illnesses, mental disorders are not solely determined by biological factors, but are influenced by an interplay of biological, psychological, and social factors (19). In this paper, we have added “environmental” to “social” recognizing the close interconnection between environmental factors and social elements. In this regard, both aspects (social and environmental) are encompassed within the same category. The comprehensive bio-psycho-socio-environmental (BPS-E) model has been documented in other sources (20) and is part of the curriculum in the course “Exploring the Relationship Between Anxiety and Depression” offered by the Open University (21).

The biological component of the model seeks to identify the influence of physiological mechanisms, such as genetic predispositions, neurological imbalances, and the overall impact of physical health, on mental well-being. The psychological component of the model pinpoints the impact of cognitive processes, emotional responses, and individual coping mechanisms on mental health. Moreover, the social-environmental component highlights the influence of interpersonal relationships, environmental factors, societal structures, and cultural influences on mental health.

The BPS-E model approach implies the importance of a holistic understanding of a person's mental health and the need for a multidisciplinary approach in health care when assessing and managing mental disorders. In this regard, our qualitative study aims aimed to provide an in-depth understanding of how biological, social-environment, and psychological factors influenced the mental health of adolescents living in Kampala, Uganda. We also aimed to understand how COVID-19 played a role in exacerbating the mental health outcomes of adolescents.

## Methods

**Figure 1:**
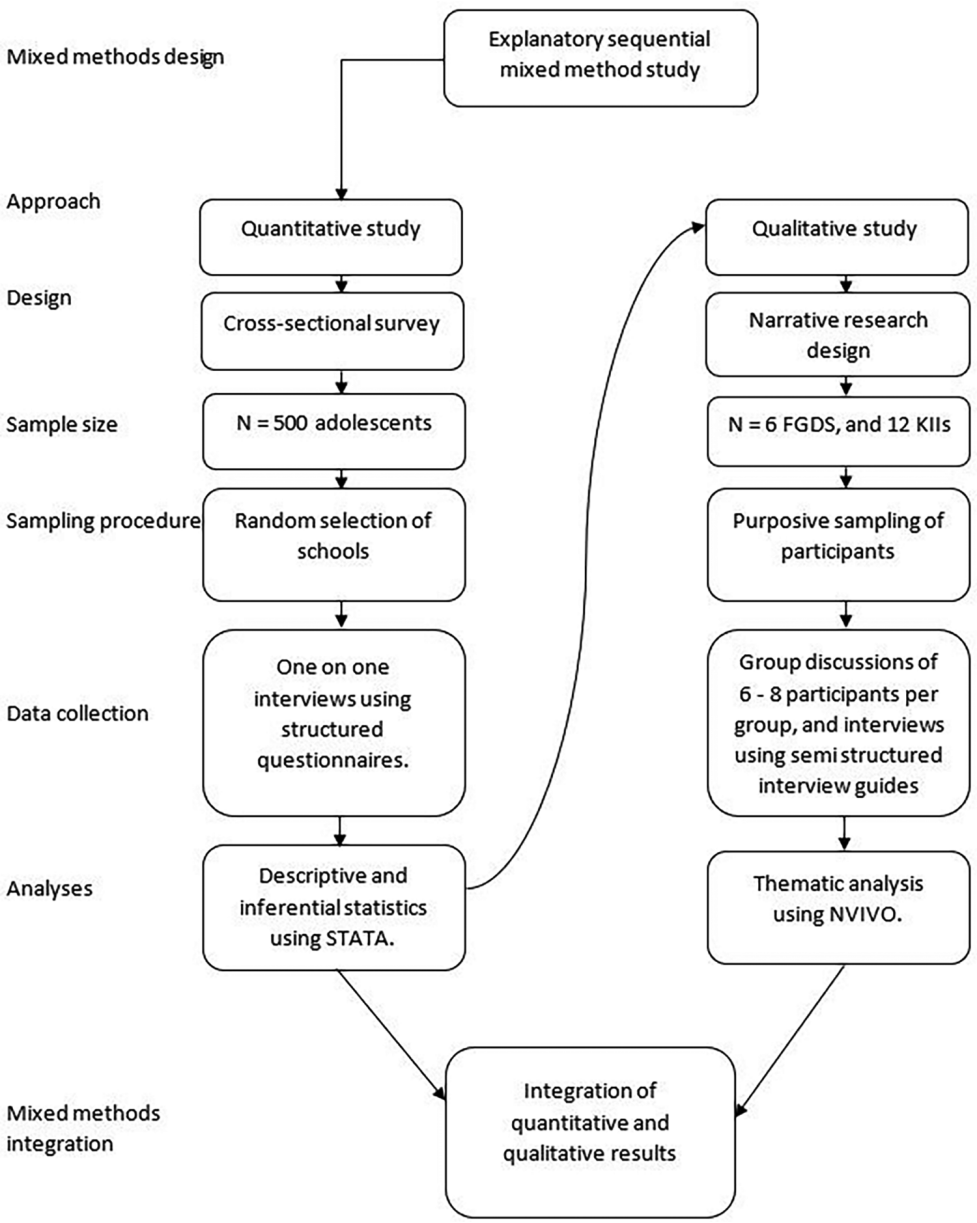
The explanatory sequential mixed-methods procedure used in the study.

This study presents the results of a formative phase part of a larger project that aimed to assess and support mental health outcomes among adolescents urban areas in Kenya and Uganda (22). The results of the formative phase from the Kenya data are reported elsewhere (4). An overview of the methodology utilized in this study is illustrated in Figure 1.

### Study design & study area

This study utilized an explanatory sequential mixed method study design. This was done by collecting quantitative data first to mainly describe the burden of mental health problems including depression and anxiety among adolescents. Subsequently, qualitative data was collected to provide deeper insights into the quantitative findings by exploring the underlying factors linked to the identified burden and contextual variations in observed trends.

The study aimed to target both school going and out-of-school adolescents aged between 13 and 19 years. Quantitative data for the study were collected from November to December 2021, whereas qualitative data collection took place between May and July 2022 in Kampala, Uganda. The study was conducted across all five (5) Divisions of Kampala City namely, Kampala Central, Kawempe, Lubaga, Makindye, and Nakawa.

### Eligibility criteria and participants' recruitment

Adolescents were included in the study if they met the following criteria:

1. Aged 13–19 years as evidenced by their birth certificates
2. Able to consent for those that were 18 years and above
3. Parents or legal guardians providing consent for adolescents below 18 years old, followed by voluntary assent from the adolescents.
4. Could speak either English or Luganda (the widely spoken language in Kampala)
5. If they were either school-going or out of school

Key informants were included if they were either teachers, parents, representatives of civil society organizations, or religious leaders. The key informants were recruited from the community bordering the selected schools and were purposively sampled.

### Sample size estimation

The total sample size required was calculated based on previous proportion estimate of 18% (23) of mental health problems among adolescents in Uganda. We used the sample size formula below for the single proportion to calculate the total sample required. This formula was used since we only considered adolescents for this study.

$$n=\frac{DEFF\cdot Z_{1-\alpha /2}^{2}\cdot p\cdot (1-p)}{d^{2}\cdot (1-NR)}$$

We considered 95% CI with a Z-score of 1.96, design effect = 2, *d* (precision or margin of error) of 0.05, and a non-response rate of 1%, resulting to a sample of 458, whereby we recruited 500 participants.

### Data collection procedures

Four enumerators were recruited and trained before the field data collection. The training was facilitated by JA, GM, MN, AM, RO, AMS, and AA. The enumerators were taken through overview of constructs, reviewing data collection tools, psychological first aid, consent, privacy, confidentiality and ethics, conducting face-to-face interviews, recruitment, mobilization strategy, COVID-19 protocols, and data management. The enumerators practiced how to obtain consent through role plays. Additionally, a pilot study was carried out to pretest the tools and data collection process. The response from the pilot study was used to refine the questionnaire and improve the study procedures.

For the school-going adolescents, we obtained the list of schools from the Kampala Capital City Authority (KCCA), and we applied simple random sampling to select a total of 10 secondary schools. Adolescents were classified as school going adolescents if they were currently registered with the selected ten schools, a confirmation provided by their selected specific educational institution. To optimize data collection during school closures, the study focused on day-school options, other than those from boarding facilities. This decision was informed by the expectation that students in boarding facilities might be more challenging to reach due to their greater distance from the schools compared to their day-schooling counterparts. The aim was to prevent participants from having to travel long distances away from the city, especially during the partial lockdown period.

Adolescents from the selected schools were mobilized from their homes through collaboration with school administrations and parents. Teachers generated lists of students aged 13–19, and a gender-stratified random selection of 30 adolescents from each school was made using the RNG® application. Selected individuals were invited to the school for full consent and interviews, with replacements made if participants couldn't attend on the specified date.

For the out-of-school adolescents, we obtained clearance from the KCCA Director for Public Health and worked closely with the village health teams, the local council 1 chairpersons, and the village health teams (VHTs) or any other delegated authority by the LC1 chairpersons' community champions to recruit participants who met our inclusion criteria. The operational definition of out-of-school adolescents was those adolescents who were not enrolled in either primary or secondary schools (24) at the time of our study. The LC1 chairpersons and VHTs were instrumental in identifying potential out-of-school adolescents in the study areas, as they had adequate knowledge and a good understanding of the study area. They would provide the data collection team with a list of potential participants, serving as the sampling frame for random selection, stratified by gender. Subsequently, selected participants would be individually invited through the area chairperson or VHT to convene at a designated location on a specified day for full consenting and data collection.

Following the completion of quantitative data collection and analysis, the subsequent phase entailed qualitative data collection. The qualitative data collection employed two data collection methods: focus group discussions (FGDs) involving a randomly selected subset of adolescents derived from the quantitative phase, and key informant interviews with teachers, parents, representatives of civil society organizations, and religious leaders who were purposively selected. The different methods were used to get a diverse range of views from participants. The FGDs were comprised of 6–8 participants. Categorization of the FGD participants by school status (either school going or out of school) and gender ensured a conducive environment to enhance free expression during the discussions.

During the data collection, all stipulated COVID-19 guidelines set by the Ugandan government and the World Health Organization were followed, and data collection was carried out in designated schools, and community sites in each division. Adolescents under eighteen years of age were accompanied by their parents or legal guardians to ensure that both consent and assent were obtained. At the end of the data collection, participants' travel costs were reimbursed.

### Data collection tools

#### Sociodemographic characteristics

We obtained information on the adolescents' gender, age, school attendance (whether they were school-going or out-of-school), level of education, religion, and household socioeconomic status using a socio-demographic survey tool developed by the project team. The assessment of household socioeconomic status involved an inquiry about the items within their homes e.g., motorcycle, radio, mobile phone etc. This evaluation utilized an assets index employed in previous studies centered on adolescents (4, 25).

#### Generalized anxiety disorder-7 (GAD-7)

We assessed the anxiety levels of the adolescents using the GAD-7 questionnaire (26). The GAD-7 is a validated tool that has been used extensively to determine the level of anxiety among individuals. The tool comprises seven items with scores on a Likert scale from 0 (not at all), 1 (several days), 2 (more than half the days), and 3 (nearly every day). The total scores range from 0 to 21 with cutoff points of 0–4, 5–9, 10–14, and 15–21 (representing none, mild, moderate, and severe anxiety respectively). Results of the psychometric analysis on the GAD-7 scale for the current study showed a high internal reliability (Cronbach's alpha = 0.81).

#### Patient health questionnaire-9 (PHQ-9)

The severity of depression among the adolescents was determined using the PHQ-9 tool (27). The PHQ-9 is a nine-item tool for measuring the severity of depression. The tool has scores on a 4-point Likert scale ranging from 0 (not at all), 1 (several days), 2 (more than half the days), and 3 (nearly every day). The total scores range from 0 to 27 with cutoff points of 0–4, 5–9, 10–14, 15–19, and 20–27 (representing none, mild, moderate, moderately severe, and severe depression respectively). The reliability test revealed a high reliability (Cronbach's alpha = 0.81).

#### Qualitative interview guides

The qualitative study employed semi-structured interview guides developed by the research team. These guides entailed questions about the general knowledge, perceptions, and attitudes toward mental health, as well as questions about COVID-19 among other aspects. For example, to understand the triggers associated with poor mental, adolescents were asked the following question during the group discussions. “*In your own opinion, what are the factors that impact negatively on adolescents' mental health?”.* To understand how the pandemic impacted the adolescents' mental health, adolescents' participants were asked: “*In what ways did the COVID-19 pandemic affect your mental health?”* Similarly, the key informants were asked similar questions, but their purpose was to delve deeper, considering their first-hand experience in interacting with adolescents.

### Data management and analysis

All quantitative data were collected using the Open Data Kit (ODK) through the study tablets. The tablets were password-protected and encrypted to ensure confidentiality and to avoid data loss and were only available to authorized personnel. The data was uploaded daily to the server for storage, which could only be accessed by the data manager.

Stata version 17 was used to analyze the quantitative data (28). The sample characteristics of the adolescents were summarized using proportions, means, and standard deviations. The differences between the in-school and out-of-school groups were investigated using the independent t-test for continuous variables and Pearson's chi-squared test for categorical variables. For depression and anxiety, the point prevalence and corresponding 95% confidence intervals (95% CI) were calculated. Figures and tables were used to present the results. Logistic regression models were used to identify factors associated with common mental health problems. Factors that had a *p*-value of <0.25 in the univariate model were fitted in the multivariate model.

For the qualitative data, the audio recordings of the FGDs and KIIs were transcribed verbatim and proofread by the team to ensure accuracy. Since the FGDs were mostly conducted in Luganda, the transcripts were translated into English, and the translation was verified by the study team. The transcripts were then imported into NVivo 14 (QSR International) software for data analysis. A thematic analysis approach was used to guide the analysis. We employed the BPS-E model, which inherently identifies three key categories linked to mental health: biological, psychological, and social-environmental factors. These three categories formed the core themes in our study. A research assistant developed codes from the transcripts, which were subsequently refined and revised by the authors (GM and AA). Codes that covered a similar subject were grouped to form sub-themes. Related sub-themes were then grouped under the three themes.

### Ethics approval and consent to participate

The ethics review for this study was obtained from The AIDS Support Organization Research and Ethics Committee (TASO-2021-18), and a research license was granted by the Uganda National Council for Science and Technology (UNCST) (Ref no: HS1939ES). Additionally, administrative clearance was obtained from the Kampala Capital City Authority (KCCA) Director for Education and Social Services, the respective headteachers, village health teams (VHTs), and the LC1 chairpersons were then approached for their further approval and administrative support during the data collection exercise. All participants provided informed written consent to participate in this study. The field activities were conducted per the relevant guidelines and regulations.

## Results

### Quantitative results

Table 1 presents the socio-demographic characteristics of participants for the quantitative data. A total of 500 participants participated in the study, in which the majority (60%) were school-going adolescents. There was an almost equal number of female and male participants (49.2% vs. 50.8%). The participants in the study had an average age of 17.1 years (*SD* = 1.7), with no significant difference between the two groups. In-school adolescents had higher household social economic status (*M* = 4.4, SD = 1.5), as compared to out-of-school adolescents (*M* = 3.1, *SD* = 1.3) *p* < 0.001. Most of the participants were Christians (77.0%), while 71.2% were in secondary schools. Furthermore, 2.0% of adolescents were parents, and 2.4% of females were pregnant, with most of them from the out-of-school group. Furthermore, the results show a significant difference in education level, religious denomination, having a child(ren), and being expectant between school-going and out-of-school adolescents.

**Table 1:** Socio-demographic characteristics of participants.

	Overall *n* (%)	In school *n* (%*)*	Out school *n* (%)	*P*-value
Overall	500	300 (60%)	200 (40%)	–
Gender				
Female	246 (49.2%)	148 (49.3%)	98 (49.0%)	0.942^†^
Male	254 (50.8%)	152 (50.7%)	102 (51.0%)	
Age mean ± SD	17.07 ± *1.67*	17.10 ± *1.51*	17.05 ± *1.89*	0.768*
Level of education				
None	3 (0.6%)	-	3 (1.5%)	**<0.001^†^**
Primary	141 (28.2%)	-	138 (69.0%)	
Senior	356 (71.2%)	300 (100.0%)	59 (29.5%)	
Religion				
Christian	385 (77.0%)	235 (78.3)	150 (75.0%)	**0.034^†^**
Islam	104 (20.8%)	55 (18.3)	49 (24.5%)	
Other e.g., traditional	11 (2.2)	10 (3.3)	1 (0.5%)	
Social Economic Status mean ± SD	3.88 ± *1.57*	4.40 ± *1.54*	3.10 ± *1.25*	**<0.001** *
Have any child(ren)				
Yes	10 (2.0%)	1 (0.3%)	9 (4.5%)	**0.001^†^**
No	490 (98.0%)	299 (99.7%)	191 (95.5%)	
Currently pregnant*^a^*				
Yes	6 (2.4%)	0	6 (6.1%)	**0.004^†^**
No	236 (95.9%)	147 (99.3%)	89 (90.8%)	
Don't know	1 (0.4%)	0	1 (1.0%)	
Missing	3 (1.2%)	1 (0.7%)	2 (2.0%)	

SD, Standard deviation.

^a^ Respondents are only girls.

* *P*-values for the continuous variable are from the student's *t*-test.

^†^ *P*-values for binary or categorical variables are from Pearson's chi-squared test.

Bold, statistically significant results (*P*-values).

Table 2 summarizes the prevalence estimates for both depressive depression and anxiety symptoms. In terms of severity for both depression and anxiety, the prevalence of the two disorders was relatively higher among out-of-school adolescents compared to in-school. Using a cut-off score ≥10 for PHQ-9, the prevalence of depression among school-going adolescents was 14.0% (95% CI: 10.5–18.4); relatively significantly lower than the out-of-school at 21.5% (95% CI: 16.3–27.8) [*p* = 0.029]. Similarly, out-of-school adolescents had a significantly higher prevalence of anxiety 17.5% (95% CI: 12.8–23.5) than in-school adolescents 13.2% (95% CI: 10.5–16.5), *p* = 0.020. The overall prevalence of positive screen for comorbidity for both depression and anxiety symptoms was 9.4% (95% CI: 7.1–12.2), with a high prevalence score in out-of-school compared to school-going adolescents (14.0% vs. 6.3%, *p* < 0.004).

**Table 2:** Prevalence of common mental disorders among school-going as compared to the out-of-school adolescents.

	Overall, *n* = 500		School going adolescents, *n* = 300		Out-of-school adolescents, *n* = 200		*P*- value
	Frequency	Prevalence (95% CI)	Frequency	Prevalence (95% CI)	Frequency	Prevalence (95% CI)	
The severity of depressive symptoms							
None (0–4)	270	54.0 (49.6–58.3)	173	57.7 (52.0–63.2)	97	48.5 (41.6–55.5)	0.094
Mild (5–9)	145	29.0 (25.2–33.1)	85	28.3 (23.5–33.7)	60	30.0 (24.0–36.8)	
Moderate (10–14)	61	12.2 (9.6–15.4)	33	11.0 (7.9–15.1)	28	14.0 (9.8–19.6)	
Moderately severe (15–19)	19	3.8 (2.4–5.9)	7	2.3 (1.1–4.8)	12	6.0 (3.4–10.3)	
Severe (20–27)	5	1.0 (0.4–2.4)	2	0.7 (0.2–2.6)	3	1.5 (0.5–4.6)	
Positive depression screen (cut-off score ≥10)							
Yes	85	17.0 (13.9–20.6)	42	14.0 (10.5–18.4)	43	21.5 (16.3–27.8)	**0.029**
The severity of anxiety symptoms							
None (0–4)	284	56.8 (52.4–61.1)	189	63.0 (57.4–68.3)	95	47.5 (40.6–54.5)	**0.006**
Mild (5–9)	150	30.0 (26.1–34.2)	80	26.7 (21.9–32.0)	70	35.0 (28.7–41.9)	
Moderate (10–14)	49	9.8 (7.5–12.7)	23	7.7 (5.1–11.3)	26	13.0 (9.0–18.5)	
Severe (15–21)	17	3.4 (2.1–5.4)	8	2.7 (1.3–5.3)	9	4.5 (2.3–8.5)	
Positive anxiety screen (cut-off score ≥ 10)							
Yes	66	13.2 (10.5–16.5)	31	10.3 (7.3–14.3)	35	17.5 (12.8–23.5)	**0.020**
Positive screen for comorbid depressive, and anxiety symptoms							
Yes	47	9.4 (7.1–12.2)	19	6.3 (4.1–9.7)	28	14.0 (9.8–19.6)	**0.004**

95% CI, 95% confidence interval; *P*-value, probability value.

Table 3 illustrates results from multivariate logistic regression model analyses assessing the correlates i.e., both risk and protective factors associated with depression and anxiety. Results of the univariate analysis can be found in Supplementary Table S1.

**Table 3:** A multivariate logistic model showing the relationships between predictor variables and depression, and anxiety.

	Depression		Anxiety	
	(OR: 95%CI)	*P*-value	(OR: 95%CI)	*P*-value
Socio-demographic				
Gender				
Male	Reference			
Female	–	–	–	–
Age	–	–	–	–
Schooling				
Yes	Reference			
No	1.90 (1.03–3.51)	**0**.**040**	0.73 (0.25–2.09)	0.554
Level of education				
None	Reference			
Primary	–	–	0.44 (0.02–11.05)	0.615
Senior	–	–	0.20 (0.01–5.73)	0.348
Religion				
Christian	Reference			
Islam	1.24 (0.60–2.57)	0.561	1.09 (0.49–2.43)	0.841
Other e.g., traditional	2.52 (0.52–12.32)	0.253	2.45 (0.39–15.22)	0.337
Social Economic Status	–	–	0.81 (0.64–1.02)	0.074
Have any child(ren)				
Yes	Reference			
No	–	–	–	–
COVID-19 related questions				
Receive support before lockdown (Psychosocial support, Support from social services, educational support)				
Yes	Reference			
No	–	–	2.43 (0.72–8.21)	0.153
Having COVID-19 infection				
No	Reference			
Yes	–	–	0.22 (0.04–1.14)	0.071
Having someone close infected with COVID-19 e.g., a family member				
No	Reference			
Yes	3.35 (1.85–6.07)	**<0**.**001**	3.50 (1.80–6.79)	**<0**.**001**
Parents and peer relationships				
Engaging with friends				
Rarely	Reference			
Occasionally	0.35 (0.14–0.84)	**0**.**019**	1.27 (0.57–2.84)	0.559
Frequently	0.23 (0.07–0.73)	**0**.**012**	1.09 (0.41–2.91)	0.867
Feeling lonely				
Not at all	Reference			
Sometimes	2.20 (1.17–4.12)	**0**.**014**	3.52 (1.75–7.10)	**<0**.**001**
Always	11.81 (4.15–33.58)	**<0**.**001**	7.54 (2.63–21.68)	**<0**.**001**
Closeness with parents				
Not very close	Reference			
Fairly close	0.32 (0.12–0.82)	**0**.**018**	0.84 (0.28–2.53)	0.756
Very close	0.54 (0.23–1.28)	0.161	1.49 (0.52–4.24)	0.454
Have conflicts in the family				
Never	Reference			
Occasionally	2.78 (1.44–5.39)	**0**.**002**	2.15 (1.03–4.51)	**0**.**043**
Frequently	1.79 (0.76–4.19)	0.181	2.36 (0.97–5.74)	0.057
Psychosocial stressors questions				
Feeling unsafe				
No	Reference			
Yes	4.88 (2.70–8.83)	**<0**.**001**	4.21 (2.22–7.98)	**<0**.**001**
Sexual abuse				
No	Reference			
Yes	–	–	2.65 (0.96–7.32)	0.061
Substance use				
No	Reference			
Yes	1.25 (0.59–2.62)	0.563	1.32 (0.61–2.86)	0.478

OR, Odds ratio; 95% CI, 95% confidence interval; *P*-value, probability value; COVID-19; Sars-cov-2 (coronavirus disease 2019).

Being out of school was the only socio-demographic found to be significantly associated with higher odds of depression (OR = 1.90, 95% CI 1.03–3.51, *p* = 0.040). Among pandemic-related factors, the only factor identified associated with higher odds of depression was having a close person infected with COVID-19 (OR = 3.35, 95% CI 1.85–6.07, *p* < 0.001). Furthermore, loneliness (OR = 11.81, 95% CI 4.15–33.58, *p* < 0.001) and intermittent familial conflicts (OR = 2.78, 95% CI 1.44–5.39, *p* = 0.002) emerged as factors associated with depression in the context of parental and peer relationships. The only psychosocial stressor identified to be significantly associated with higher odds of depression was the feeling of being unsafe (OR = 4.88, 95% CI 2.70–8.83, *p* < 0.001). Two factors were identified as associated with lower odds of depression (protective factors): frequently engaging with friends (OR = 0.23, 95% CI 0.07–0.73, *p* = 0.012) and having a close relationship with parents (OR = 0.32, 95% CI 0.12–0.82, *p* < 0.001).

In terms of anxiety, no socio-demographic factor was identified as significantly associated with higher odds of anxiety. Having someone close infected with COVID-19 (OR = 3.50, 95% CI 1.80–6.79, *p* < 0.001) emerged as the only factor identified with higher odds of anxiety in the context of the pandemic factors. Two factors about parents and peer relationships were identified to be associated with high odds of anxiety: having conflicts in the family (OR = 2.15, 95% CI 1.03–4.51, *p* = 0.043), and loneliness (OR = 7.54, 95% CI 2.63–21.68, *p* < 0.001). Additionally, the only psychosocial stressor identified to be significantly associated with higher odds of anxiety was the feeling of being unsafe (OR = 4.21, 95% CI 2.22–7.98, *p* < 0.001). There was no significant factor associated with lower odds of anxiety.

### Qualitative results

Table 4 presents an overview of the study's participants involved in the qualitative study. In this phase, a total of 6 Focus Group Discussions (FGDs) were undertaken. Additionally, the study included 12 Key Informant Interviews (KIIs). Overall, the qualitative data collection phase encompassed a total participation of 50 individuals.

**Table 4:** Breakdown of the FGDs and KIIs.

Focus group discussions	
Category	Breakdown
Male adolescents	3 FGDs (2 out-of-school,1 school-going)
Female adolescents	3 FGDs (1 out-of-school, 2 school-going)
Total	6 FGDs (3 out-of-school, 3 school-going)
Key informant interviews	
Teachers	3 (2 male, 1 female)
Religious leaders	4 (2 female, 2 male)
Civil society organizations	2 (1 male, 1 female)
Parents	3 (2 female, 1 male)
Total	12 (6 female, 6 male)

FGDs, focus group discussions.

### Factors associated with poor mental health among adolescents

**Figure 2:**
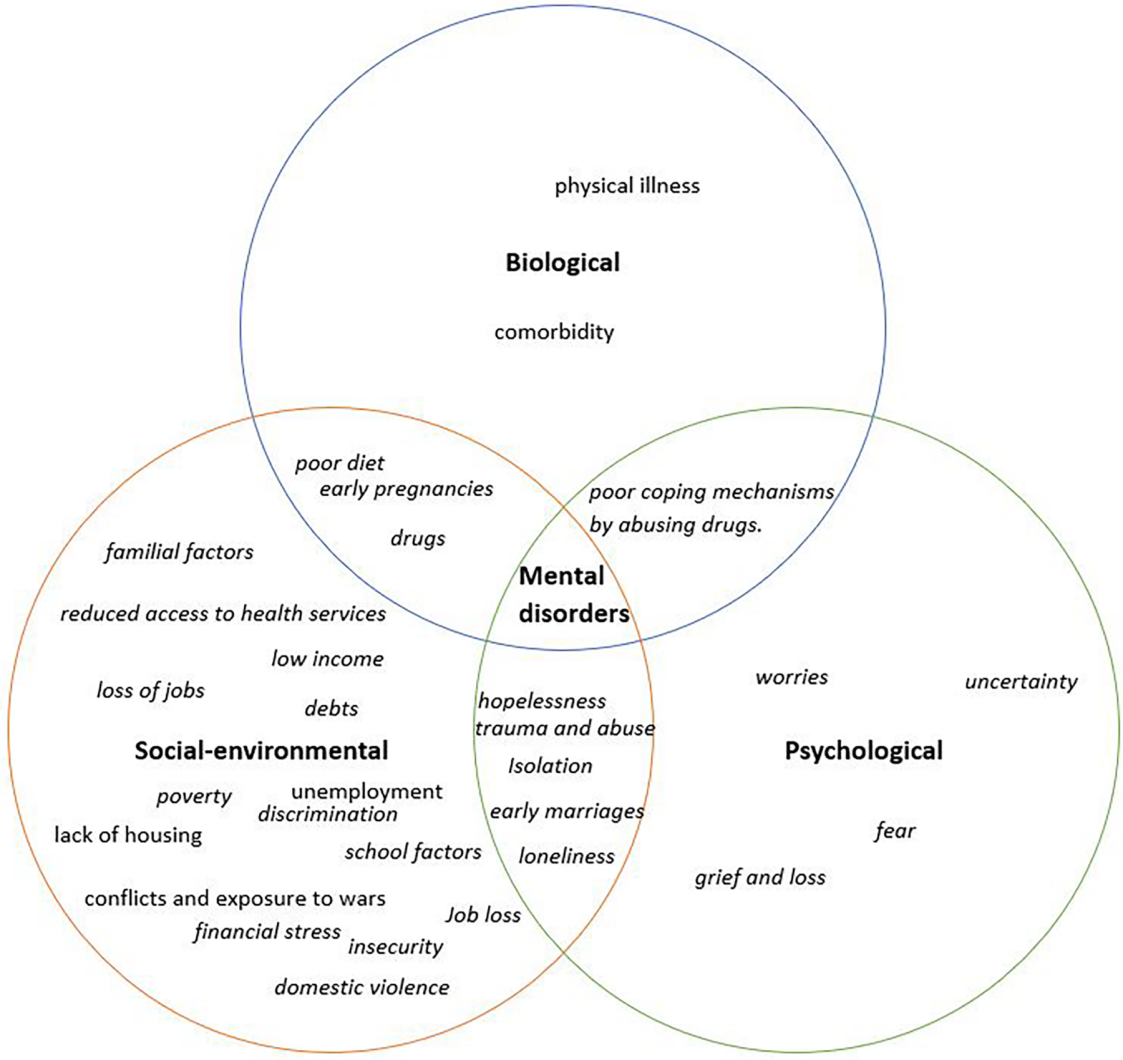
Factors identified to be associated with mental health problems: factors in italics are the ones noted to have been exacerbated by COVID-19.

Through the qualitative study, we were able to identify and pinpoint the main factors associated with poor mental health. Figure 2 illustrates the three themes and the associated factors that emerged as triggers of mental health problems. Furthermore, for a more comprehensive understanding, Supplementary Table S2 offers a detailed breakdown of these categories along with their respective sub-themes, as well as providing examples of key quotes and their frequencies.

### Biological factors

The qualitative analysis identified a number of biological factors associated with poor mental health as shown in Figure 2. This includes physical illness, comorbidity, absence of food, and substance abuse. Furthermore, pregnancies, poor diet, and substance abuse cuts across not only biological but as socio-environmental factors.

Among the physical illnesses that were mentioned were conditions like HIV/AIDS, COVID-19, and tuberculosis. Particularly, the participants noted how stigma and discrimination often linked with these diseases can result in depression, ultimately escalating to thoughts of suicide.

“Health issues also play a significant role. For instance, when adolescents contract diseases such as tuberculosis or HIV/AIDS and others become aware of it, the person often experiences a sense of depression due to the stigma they encounter. For example, providing a separate cup or plate for someone with tuberculosis. This discrimination can become so severe that it leads a person to contemplate suicide.” *(FGD female out-of-school adolescent)*

The drugs predominantly identified as being abused by adolescents were marijuana and alcohol. It was observed that drug use served not only as a contributor to mental health problems but also as a strategy employed by some adolescents to cope with existing mental health issues. Drug use among adolescents was primarily attributed to negative influences from peers, media, and role models. Additionally, unemployment emerged as a contributing factor to drug use, particularly among out-of-school adolescents.

“Among the things that cause suicidal thoughts among adolescents, we go back to the abuse of drugs…” *(KII male parent)*

### Psychological factors

Several psychological factors were identified as contributors to mental health problems among adolescents. Key psychological factors include hopelessness, and worries.

Adolescents pointed out that being coerced into early marriages, experiencing heartbreaks in relationships, and the fear of neglect and ridicule following a pregnancy could result in poor mental health outcomes.

“Being forced into early marriages. Some parents may force their children to join early marriages and instead of the children getting married to old men, some choose to take their own life.” *(FGD female school-going adolescent)*

Child abuse and maltreatment, encompassing sexual abuse, physical abuse, and emotional abuse, constituted additional psychological factors that were identified. Participants noted that girls were predominantly the victims of rape and defilement. In addition, some male participants noted that some boys experience abuse, for instance, one participant highlighted a growing prevalence of boys experiencing sodomy.

Within the context of physical abuse, participants emphasized that instances mostly occurred within families, often involving parents mistreating e.g., physical abuse of their children. Regarding emotional abuse, participants highlighted instances of school-going adolescents being mistreated by their teachers, which significantly impacted their mental well-being. A particularly concerning revelation came from one participant who mentioned instances of foreign students being subjected to abuse by a teacher.

“You find a teacher being so strict, for example towards foreign students. The teacher will provoke them, and it ends up looking like segregation which leads to mental health issues. Considering they are in a country that is not their own, this ultimately affects their minds.” *(FGD male school-going adolescent)*

### Social-environmental factors

Through the analysis, various social-environmental factors that lead to mental health problems among adolescents were identified. These factors include poverty, unemployment, financial constraints, low income, and school-related factors.

Participants highlighted that adolescents hailing from poor families often struggle with meeting basic needs like food, rent, and school fees, which consequently lead to heightened stress. Poverty was further linked to family conflicts, pushing adolescents towards adopting harmful coping mechanisms such as engaging in prostitution, theft, and drug use, amplifying their distress. Increased responsibility coupled with lack of jobs, and low income stood out as a driver of mental health problems among out-of-school adolescents.

Most participants highlighted that isolation, loneliness, and lack of social support constituted significant social factors linked to poor mental well-being. The participants noted that society at large plays a role as well in exacerbating adolescents' mental health problems, as young people are left alone to navigate their challenges without adequate support.

“What I can say is that the societies we live in also contributes to adolescents' having mental problems. Because when you are staying somewhere and you just see people around you, yet no one can help you in case you get a problem. This brings about mental challenges …” *(FGD male out-of-school adolescent)*

Additionally, it was noted that adolescents are day-by-day becoming disconnected from the world around them, including their community, schools, religion, and family. This disconnection was noted to contribute to isolation and loneliness.

School-related factors that were identified to result in mental health problems include, bullying, failure of exams, financial constraints such as the inability to afford school fees, overwhelming academic pressures, and lack of sanitary towels for school-going girls. It was noted that these challenges often led to some adolescents dropping out of school. One adolescent provided insight into the impact of the lack of school fees, sharing how this circumstance led her to abandon her aspirations of becoming a nurse.

“I wanted to become a nurse, but unfortunately, I couldn't achieve that dream. I was passionate about education, but due to my circumstances with a single mother who couldn't afford school fees, I faced obstacles. I'm determined not to let my children face the same situation. This experience posed mental challenges for me, even leading me to leave home” *(FGD female out-of-school adolescent)*

### The role COVID-19 played in exacerbating mental health issues

Participants emphasized the profound impact of the COVID-19 pandemic and the associated containment measures on the mental well-being of adolescents. COVID-19 was noted to have exacerbated most of the identified biological, socio-environmental, and psychological factors.

For example, the pandemic was noted to have brought about financial strain. The account below is from an adolescent who shared how his father had to make a difficult decision by asking his younger siblings to temporarily drop out of school due to financial constraints after the lockdown, as he could only afford to pay the school fees for one person.

“Some of the worries are that some parents were used to having money before lockdown and now after they locked down everything, it led to depression which made families unstable. Like my father now, he has three children and before lockdown, he could afford to pay school fees for all of us but now he finds it difficult to pay it all. So, he told the young ones to first drop out for me to finish my studies” *(FGD male school-going adolescent)*

Additionally, the pandemic is noted to have brought about uncertainty, worries, and fear; feelings of isolation and loneliness; financial strain; experiences of grief and loss; and domestic and gender-based violence.

The worries and fears centered around fear of contracting the disease, adolescents being worried about the well-being of their families, school-going adolescents worrying whether they will ever go back to school again, and uncertainty about their future. It was observed that some adolescents, upon losing their parents or close family relations, experienced profound hopelessness.

The following account is from an adolescent who narrated how his friend's mental well-being was negatively affected after the loss of his grandmother.

“My friend lost his grandmother, and he experienced a significant mental breakdown. As a result, he was admitted to a mental health unit to receive counseling…” *(FGD male school-going adolescent)*

It was observed that certain measures, such as lockdowns and movement restrictions, posed challenges for individuals in accessing healthcare services. The following account is from an out-of-school adolescent highlighting how she was affected.

“I was much affected because I was always stressed because I was pregnant, and it made me too sick, but I couldn't go to the hospital. Even on the day I was to give birth, I got labour pains at night but there was no boda-boda [commercial motorcycle] that you would find on the way. I was scared that my child was going to die because I went to the hospital in the morning because of a lack of means of transport. Another thing, our people died but we couldn't go to bury them, it affected us badly that we were stressed because of the situation” *(FGD female out of school adolescent)*

The pandemic and its impact led to adolescents developing poor coping mechanisms, such as resorting to drug use, while others became entangled in early marriages as exemplified below. However, it was noted not all adolescents resulted in poor coping mechanisms, as others started looking for jobs, to take care of themselves and their families.

“Some adolescents looked at the world ending so they lost hope, they developed more stress than before, they involved themselves in early marriages and more alcohol abuse while others developed suicidal thoughts and some even lost their lives.” *(KII female teacher)*

## Discussion

This study provides crucial insights into the mental well-being of adolescents living in urban context in the Uganda's capital in the context of COVID-19. Notably, the observed prevalence for depression and anxiety stood at 17.0% and 13.2% respectively. However, there is paucity of studies examining mental health outcomes among adolescents in Uganda, resulting in fewer comparative studies done during the pandemic within Uganda. Nevertheless, in comparison to regional studies conducted in sub-Saharan Africa during the pandemic, there exists significant heterogeneity in estimates, with a majority of studies indicated prevalence estimates for depressive and anxiety disorders ranging between 10% and 40% (4, 5, 29, 30).

Our reported prevalence is notably higher among out-of-school adolescents compared to their schooling peers. This trend was as well observed in the sister study conducted in Kenya (4). The challenges out-of-school adolescents face in trying to meet basic needs make them more prone to poor mental health. Schools hold a pivotal role in enhancing mental well-being and overall student welfare. Teachers' support, co-curricular activities such as games, and peer support within schools are aspects that play a crucial role in supporting adolescents with mental disorders. Notably, out-of-school adolescents lack access to these essential forms of support, which potentially contributes to their disproportionately greater burden of mental health problems when compared with their school-attending counterparts (4).

Physical illness was a key biological factor identified associated with mental health problems in the qualitative study. The stress that is associated with chronic illnesses such as untreated unresolved tuberculosis can render patients more susceptible to mental health disorders. The relationship between physical illness and mental health problems has been studied extensively and a strong link has been found between the two (31, 32). For example, a systematic review of the epidemiology of mental disorders revealed that the onset of emotional disorders among children and adolescents was highly predicted by having a physical illness (33). Additionally, discrimination and stigma that are associated with some physical conditions can contribute to heightened distress, potentially escalating to suicidal ideation. Substance abuse was another key factor identified linked to depression and anxiety. Numerous studies have reported that during adolescence, engaging in various substance-use behaviours including alcohol consumption often increases the risk of depressive symptoms among adolescents (4, 34). The pandemic also placed adolescents at a higher risk for engaging in risky behaviors, including increased drug use. A recent study exploring substance use before and during the pandemic among Uganda adolescents reported an increase in use during the pandemic; however, this change wasn't statistically significant when compared to the pre-pandemic period (35).

We identified a couple of social-environmental factors linked to poor mental health among adolescents. The key factors include poverty, unemployment particularly among out-of-school adolescents, social isolation, and adolescents playing caregiving roles. Previous studies have studied the link between socio-economic factors and mental health (36, 37). A global systematic review established that a low socioeconomic status was highly correlated to higher rates of mental health problems in childhood and adolescence (38). Given the well-established impact of poverty on exacerbating mental health problems, implementing poverty alleviation initiatives such as the provision of grants and un/conditional cash transfers, providing materialistic school support, and enhancing food and water security in informal settlements have the potential to significantly enhance mental health outcomes (39, 40). It was noted that some adolescents had to drop out of school due to excessive caregiving responsibilities. These excessive responsibilities including caring for younger siblings, due to parents absconding their duties, made the adolescents experience heightened strain. Prior evidence has shown that the impact of adolescents' exposure to extreme adversities and constraints due to social inequalities persists well into adulthood and can have a long-term effect (41). This indicates the critical need for prevention and intervention strategies targeting early adverse experiences.

Threats to safety, violence exposure, and displacement induce psychological distress, potentially leading to various mental health challenges (42). Limited access to essentials like food and housing is another crucial factor that we identified in the study. Inadequate living conditions and insufficient nutrition heighten stress and anxiety, straining psychological well-being and exacerbating mental health issues (43). Poor working conditions were also a key factor noted particularly among the out-of-school adolescents. It was highlighted that adolescents face unfavorable environments and demanding work, as they strive to provide and take care of their families, resulting in heightened stress. These factors highlight the complex interplay between external circumstances and mental health.

The COVID-19 pandemic and its associated containment measures appear to have adversely affected adolescents' mental well-being. Loneliness was a significant factor associated with both depression and anxiety. Loneliness appears to have had a pronounced impact on adolescents, unintentionally arising as the result of the measures put in place to contain the pandemic (4). Being confined to one's house can disrupt sleep patterns and exercise habits, whereas excessive use of technology e.g., excessive use of mobile phone, and the internet resulting due to loneliness can influence a person's mental health (44). Furthermore, losing connections with others and feeling alienated and alone can result in feeling distressed and depressed (4). This emphasizes the significance of fostering healthy connections and engagement within the support systems accessible to adolescents. For instance, maintaining strong connections to family, religion, school, community, and culture is essential to ensure that adolescents do not find themselves isolated. Our study notes that the extended school closures and economic hardships led to a concerning rise in pregnancies among girls and adolescents getting entangled in early marriages. The study has shown that girls and boys reported increased tension in their households resulting in cases of emotional, physical, and sexual violence. This violence subjected to adolescents because of the pandemic had a huge toll on their mental health. Consequently, some adolescents resorted to poor coping mechanisms, including substance abuse. The adverse consequences brought about by the pandemic highlight the importance of safeguarding adolescents' mental well-being, primarily by addressing the diverse challenges they face.

### Comparing results from quantitative and qualitative analyses

Both the quantitative and qualitative studies identified similar factors that significantly contribute to mental health problems among adolescents. Predominant factors identified in both studies include loneliness, being out of school, and conflicts with parents. In addition, both studies highlighted the profound impact COVID-19 had in exacerbating mental health issues. Evidently, the consistency in the findings underscores the undeniable influence of these factors on adolescents' mental well-being.

### Strengths and limitations

This study has strengths and limitations. First, it not only focused on school-going adolescents but also adolescents out-of-school, a group often overlooked despite being at a high risk of mental health issues. Another strength of the study is its ability to explore an important research theme, assessing the psychological well-being and burden of mental health problems among adolescents in urban contexts Furthermore, this study used measures that have been psychometrically tested and adapted to local contexts and have demonstrated good internal consistency and validity. The fact that there was a Kenyan site was another strength of the study in terms of contextual comparisons. The use of mixed methods, in which the quantitative study informed the conceptualization of the qualitative study, enhances the overall robustness of the study. Additionally, conducting data collection during the pandemic was another strength of the study as this minimized recall bias. We believe that the triangulation approach, involving data collection from various perspectives such as adolescents, parents, religious leaders, and civil society, further strengthens the depth and reliability of the research findings.

In terms of the limitations of the study, data on the outcomes and the correlates were collected based on self-report, which is prone to information bias and the potential of recall bias. We didn't collect pre-pandemic comparison data to contextualize the quantitative data. We acknowledge that our findings may not be generalizable to the entire Ugandan population. The cross-sectional nature of the study means we cannot establish the causal relationship between the factors reported and the mental health outcomes. Nonetheless, the robustness and comprehensiveness of the study provide crucial perspectives regarding how disruptions because of the COVID-19 impacted on the mental health of adolescents living in Uganda's urban informal settlements of Kampala capital city.

## Conclusion

This study investigates the mental well-being of adolescents in Ugandan urban informal settlements of Kampala capital city in the context of COVID-19. Depression and anxiety prevalence stood at 17.0% and 13.2%, mirroring global estimates. Out-of-school adolescents had higher rates, underscoring the role of schools in supporting adolescents' mental well-being. Key factors identified through the qualitative study associated with common mental disorders include physical illnesses, substance abuse, poverty, unemployment, and isolation.

In addition, the quantitative study identified several key factors linked to poor mental health, such as being out of school, experiencing conflicts with parents, and feelings of loneliness. It was noted that the pandemic exacerbated the mental health problems of adolescents, mainly due to the containment measures that were put in place that enhanced loneliness and isolation. School closures and economic struggles led to increased pregnancies, early marriages, and household tensions, resulting in violence against adolescents. The findings stress the need for comprehensive interventions involving schools, families, communities, and policies to address these challenges and safeguard adolescent mental health, especially in marginalized urban settings. By investing in and prioritizing the mental well-being of Ugandan adolescents, we can help build a brighter future for the younger generation and ensure their long-term resilience and success.

## Data Availability

The raw data supporting the conclusions of this article will be made available by the authors, without undue reservation.
