# Supplementary material for: Exploring adolescents' mental health in Kampala, Uganda in the context of COVID-19: a mixed methods study
Source: Front Child Adolesc Psychiatry. 2025 Feb 24;4:1419043. doi: 10.3389/frcha.2025.1419043 (PMC11891163; doi:10.3389/frcha.2025.1419043)
Supplement: Supplementary file 1 [file Table1.docx]

**Supplementary table 1. A univariate logistic model showing the relationships between predictor variables and depression, and anxiety.**

|  | Depression | | Anxiety | |
| --- | --- | --- | --- | --- |
|  | (OR:95%CI) | *P*-value | (OR:95%CI) | *P*-value |
| Socio-demographic | | | | |
| Sex |  |  |  |  |
| Male | *Reference* |  |  |  |
| Female | 0.95 (0.60-1.52) | 0.845 | 1.04 (0.62-1.74) | 0.889 |
| Age | 1.05 (0.91-1.21) | 0.526 | 0.98 (0.84-1.14) | 0.766 |
| Schooling |  |  |  |  |
| Yes | *Reference* |  |  |  |
| No | 1.68 (1.05-2.69) | **0.030** | 1.84 (1.09-3.10) | **0.022** |
| Level of education |  |  |  |  |
| None | *Reference* |  |  |  |
| Primary | 0.66 (0.06-7.51) | 0.738 | 0.52 (0.05-5.91) | 0.596 |
| Senior | 0.32 (0.03-3.59) | 0.355 | 0.23 (0.02-2.54) | 0.228 |
| Religion |  |  |  |  |
| Christian | *Reference* |  |  |  |
| Islam | 0.98 (0.55-1.76) | 0.946 | 0.96 (0.50-1.84) | 0.895 |
| Other e.g. traditional | 2.87 (0.82-10.08) | 0.1o1 | 2.51 (0.65-9.79) | 0.184 |
| Social Economic Status | 0.98 (0.84-1.14) | 0.793 | 0.80 (0.67-0.96) | **0.016** |
| Have any child(ren) |  |  |  |  |
| Yes | *Reference* |  |  |  |
| No | 1.23 (0.26-5.88) | 0.799 | 0.73 (0.10-5.83) | 0.764 |
| COVID-19 related questions | | | | |
| Receive support before lockdown (Psychosocial support, Support from social services, educational support) |  |  |  |  |
| Yes | *Reference* |  |  |  |
| No | 0.90 (0.45-1.82) | 0.775 | 1.76 (0.68-4.61) | 0.239 |
| Having COVID-19 infection |  |  |  |  |
| No | *Reference* |  |  |  |
| Yes | 1.05 (0.42-2.62) | 0.917 | 0.39 (0.09-1.68) | 0.207 |
| Having someone close infected with COVID-19 e.g. a family member |  |  |  |  |
| No | *Reference* |  |  |  |
| Yes | 3.05 (1.89-4.92) | **<0.001** | 2.49 (1.48-4.21) | **0.001** |
| Parents and peer relationships | | | | |
| Engaging with friends |  |  |  |  |
| Rarely | *Reference* |  |  |  |
| Occasionally | 0.38 (0.18-0.78) | **0.009** | 0.87 (0.44-1.72) | 0.693 |
| Frequently | 0.19 (0.07-0.53) | **0.002** | 0.58 (0.25-1.34) | 0.200 |
| Feeling lonely |  |  |  |  |
| Not at all | *Reference* |  |  |  |
| Sometimes | 3.75 (2.19-6.42) | <**0.001** | 4.64 (2.51-8.56) | **<0.001** |
| Always | 18.26 (7.77-42.92) | **<0.001** | 13.72 (5.69-33.08) | **<0.001** |
| Closeness with parents |  |  |  |  |
| Not very close | *Reference* |  |  |  |
| Fairly close | 0.44 (0.21-0.89) | **0.023** | 0.65 (0.28-1.50) | 0.314 |
| Very close | 0.34 (0.17-0.66) | **0.001** | 0.58 (0.27-1.25) | 0.163 |
| Have conflicts in the family |  |  |  |  |
| Never | *Reference* |  |  |  |
| Occasionally | 3.20 (1.87-5.48) | **<0.001** | 2.56 (1.41-4.65) | 0**.002** |
| Frequently | 3.56 (1.84-6.87) | **<0.001** | 3.51 (1.73-7.09) | **<0.001** |
| Psychosocial stressors questions |  |  |  |  |
| Feeling unsafe |  |  |  |  |
| No | *Reference* |  |  |  |
| Yes | 5.71 (3.44-9.50) | **<0.001** | 5.78 (3.27-10.24) | **<0.001** |
| Sexual abuse |  |  |  |  |
| No | *Reference* |  |  |  |
| Yes | 1.46 (0.61-3.51) | 0.396 | 3.51 (1.57-7.84) | **0.002** |
| Substance use |  |  |  |  |
| No | *Reference* |  |  |  |
| Yes | 2.19 (1.25-3.84) | **0.006** | 2.29 (1.25-4.20) | **0.007** |
