# Supplementary material for: Exploring adolescents' mental health in Kampala, Uganda in the context of COVID-19: a mixed methods study
Source: Front Child Adolesc Psychiatry. 2025 Feb 24;4:1419043. doi: 10.3389/frcha.2025.1419043 (PMC11891163; doi:10.3389/frcha.2025.1419043)
Supplement: Supplementary file 2 [file Table2.docx]

| **Supplementary table 2: Themes, subs-themes, and main quotes of the qualitative analysis** | | | | |
| --- | --- | --- | --- | --- |
| **Theme** | **Key sub-themes** | **Child node** | **No. of quotes** | ***Key quotes*** |
| 1. Biological factors | 1. Physical illness | - | 4 | *“Serious diseases like HIV/AIDS. I had a cousin sister who got AIDS and at the same time got pregnant at eighteen years. After getting done, she gave birth and the child died later, so they got her going to kill herself too.”* ***(FGD Female School-going: Transcript 2)*** |
|  | 1. Drug or alcohol abuse and addiction | - Drug use caused by bad influence from  1. Peers 2. Media 3. Role models | 21 | *“Among the things that cause suicidal thoughts among adolescents, we go back to the abuse of drugs. Now that I have stayed mostly with the adolescents, you see how someone was reasoning before abusing drugs and the way he reasons after abusing drugs you might think they are two different people, so the other one who abuses drugs, makes decisions so fast even though it’s wrong, for him, he sees it to be correct. Because if he gets a small challenge, what comes to his mind is to commit suicide because he sees that’s the right thing available.”* ***(KII Male Parent: Transcript 9)*** |
|  |  | - Drug use caused by unemployment |  |  |
| 1. Psychological factors | 1. Familial factors | - Divorce and separation | 3 | *“Divorce among parents. when your parents’ divorce, children may lack parental love and they may fail to talk to someone else about what they lack and this may also cause mental problems.”* ***(FGD Female School-going: Transcript 2)*** |
|  |  | - Domestic violence | 3 | *“*we *spend our lives in domestic violence and fighting and what, this torture is not only affecting us, but our children are becoming victim.”****(KII Teacher female: Transcript 11)*** |
|  |  | - Loss of parents | 8 | *“*In *a situation when he or she loses someone, for example in the past lockdown, my friend lost his grandmother and he was running mad, there he was taken to a mental health unit for counselling.”* ***(FGD Male School-going: Transcript 1)*** |
|  |  | - Lack of responsive care | 36 | *“Some adolescents, especially girls are tortured by their stepmothers, and they develop hatred on themselves and may decide on committing suicide.* ***(FGD Male School-going: Transcript 1)*** |
|  | 1. Early pregnancies and parenthood | - Early parenthood and marriages | 2 | *“*Being *forced into early marriages. Some parents may force their children to join the early marriages and instead of the children getting married to old men, they decide to kill themselves.”* ***(FGD Female School-going: Transcript 2)*** |
|  |  | - Teenage pregnancies | 4 | *“In schools, when a girl gets pregnant and thinks of how going back home might be, they might go and commit suicide in fear of being neglected and laughed at.”****(FGD Female School-going: Transcript 2)*** |
|  |  | - Early relationships | 6 | *“Some adolescents are devoted to relationships for example between girls and boys and when they are disappointed, the feel like committing suicide”****(FGD Male School-going: Transcript 1)*** |
|  | 1. Child abuse and maltreatment | - Sexual abuse | 6 | *“* *I think the cause of mental health is being raped like most especially girls they are so affected about that thing”* ***(FGD Female School-going: Transcript 18)*** |
|  |  | - Physical abuse | 3 | *“* *Also in line with that we have got family background, someone is an orphan, has grown up in a step family, so the torture in case that family is not behaving the right way in the real growth and development, it may also be a serious challenge to the youth”* ***(KII Religious Leader: Transcript 11)*** |
|  |  | - Emotional abuse | 11 | *“you find a teacher being so strict for example on a Somali or Sudanese that she will provoke him and end up looking like segregation which leads to mental health yet they are in country that is not theirs and it affects their minds.”* ***(FGD Male School-going: Transcript 1)*** |
| 1. Social-environmental factors | 1. Poverty | - | 24 | *“I think poverty also causes them to have mental health problems. You know someone there, they are not fed well... The child keeps there all the times worried of what to eat, what to wear.”* ***(KII Parent: Transcript 10)*** |
|  | 1. Unemployment | - | 10 | *“* *I think depression and overthinking, like among some boys who impregnated girls at young age during COVID pandemic, girls were chased away from homes and now the girl had to go the boy’s house and you find the boy is not working, the boy becomes so stressed up, no working, the girl is now there, he now overthinks and thinks that the solution is now committing suicide.”* ***(FGD Female School-going: Transcript 18)*** |
|  | 1. Social isolation, lack of social support, and loneliness | - | 25 | *“What I can say is that the societies we live in also contributes to adolescents having mental problems because there is when you are staying somewhere and you see people around you, no one can help you in case you get a problem, this brings you mental challenges and you end up doing a job you were not supposed to do.”* ***(FGD Male Out-of-School: Transcript 5)*** |
|  | 1. Debts, low income | - | 3 | *“I think people start creating unnecessary debts and fail to pay them and they decide to kill themselves”* ***(FGD Male Out-of-School: Transcript no.17)*** |
|  | 1. School related factors | - Bullying | 10 | *“Bullying can also cause mental problems, because when you are bullied at school, and you feel like you are alone, nobody cares about you, you can be mentally disturbed.”****(FGD Female School-going: Transcript 2)*** |
|  |  | - Lack of school fees | 3 |  |
|  |  |  |  | *“* *To me before covid, they used not to send me back home for money everytime according to these prices, due to the increase in prices, there is no money and they now keep on chasing me to look for money yet it’s not there for school fees and there are students who get tired of being chased back home for school fees so they decide to join peer groups and take some drugs because they can’t stay home since they are also tired of being chased from school.”* ***(FGD Female School-going: Transcript 2)*** |
|  | 1. Work related factors | - | 4 | *“* *Being forced to do something. For example, at work, they might force you to do something you don’t want like lifting heavy things and it brings you desperacy and you feel like doing something and hurt yourself.”* ***(FGD Male Out-of-school: Transcript 5)*** |
|  | 1. Food insecurity | - | 5 | *“* *For example, I got a partner during covid 19 and we gave birth. The challenges I had, food was always hard to get at my young age and the man had run away. I was depressed and got mental challenges that I got bad thoughts also. I think what we go through can lead you to a wrong decision and it leads to a mental illness”* ***(FGD Female Out-of-school: Transcript 16)*** |
|  | 1. Lack of housing and properties | - | 3 | *“* *I had an experience and thought of killing myself. I had left my property at home and went to look for jobs but when I came back, I found everything was sold by my parents. I had suicidal thoughts because I had no where to start from.”* ***(FGD Male Out-of-school: Transcript 17)*** |
| 1. How COVID-19 impacted mental health of adolescents | - 1. Grief and loss | - | 6 | *“my friend lost his grandmother and he was running mad, there he was taken to a mental health unit for counselling.”* ***(FGD male school-going: Transcript 1)***  *“ Adolescents were scared and worried of not finishing school and started overthinking what they would have to do with their lives. They lost their friend through the pandemic as some left schools and got married and others lost their lives due to the corona virus.”* ***(KII religious leader: Transcript 13)*** |
|  | - 1. Financial stress | - | 8 | *“Some of the worries are that, some parents were used to having money before lockdown and now after they locked down everything, it led to depression which made families unstable like my father now, he has three children and before lockdown, he could afford paying school fees for all of us but now he finds it difficult to pay it all. So he told the young ones to first drop out for me to finish my studies.”* ***(FGD male school-going: Transcript 1)*** |
|  | - 1. Domestic violence | - | 4 | *“They were affected because there was no way of controlling their emotions. Others ended up killing themselves for example the fights that came from both the adolescents and parents created that status of hatred and in so doing, they ended up fighting, like because of the movement restrictions put up by the parents. They all got tired of themselves.”* ***(KII female teacher: Transcript 4)*** |
|  | - 1. Loss of jobs | - | 3 | *“For me, it affected me like I said, my mother lost her job and I am still stressed about the school fees and I am in senior six.”* ***(FGD male school going adolescents: Transcript 1)*** |
|  | - 1. Uncertainty, worries, and fear | - | 19 | *“There are very many worries which have come up due to lockdown because I am hearing many adolescents and parents asking where the school fees will come from, they are worried about many things, what they are going to use at school. Before, many of them did not think of it because many parents were working but now they don’t have stable jobs and everyone is worried about what they will use at school and that’s one of the worries present today to the students mainly.”* ***(FGD male school going: Transcript 1)***  *“Of course there was worry, too much stress, of course when you are not doing anything there is that kind of fear. You can’t do this because it was affected by covid, then the rules and regulations of covid so that was a terrible time where each person especially the adolescents were in total fear.”* ***(KII male teacher: Transcript 3)*** |
|  | - 1. Hopelessness | - | 4 | *“First of all, those who were in school lost hope of accessing life, they were stressed up. Their psychological minds were affected since they could not access the services like going to school on daily basis, they lost that hope, they were stressed up, some of them were hit by poverty because some students are target workers; they come to study and after go to earn something and during that time, they could not access anything.”* ***(KII male teacher: Transcript 3)***  *“ Some adolescents looked at the world ending so they lost hope, they developed more stress than before, they involved themselves in early marriages and more alcohol abuse while others developed those suicidal thoughts and some even lost their lives.”* ***(KII female teacher: Transcript 4)*** |
|  | - 1. Isolation and loneliness | - | 8 | *‘They were affected. They were very much affected, especially they lost the social connections, some of them they slide back as far as education is concerned, their mental kind of, their capability to grasp because they had spent a lot of time at home so that disturbed them, so even now teachers to make them cope, and some of them were traumatized, some of them had a lot of trauma.”****(KII religious leader: Transcript 12)***  *“It affected their social aspect of life because young people are good at socializing with parents, with neighbors, so lockdown brought in a lot of loneliness among the young people, fear, such are the things I think.”****(KII religious leader: Transcript 12)***  *“The young adolescents most of them had never seen such catastrophe, the pandemic so the isolation at home, the loneliness, the fear of what would come and the anxiety also, the depression as most of them were depressed so all brought about those suicidal tendencies and lack of proper communication.”* ***(KII religious leader: Transcript 12)*** |
|  | - 1. Reduced access to health services | - | 3 | *“ It affected them in that some health centers closed and there was no way of getting health services. Parents also lacked money and transport to take their wanting mental patients for health care services such as treatment.”* ***(KII female teacher: Transcript 4)***  *“I was much affected because I was always stressed because I was pregnant and it made me too sick but I couldn’t go to the hospital. Even on the day I was to give birth, I got labour pains at night but there was no boda boda [commercial motorcycle] that you would find on the way. I was scared that my child was going to die because I went to the hospital in the morning because of lack of means of transport. Another thing, our people died but we couldn’t go to bury them, it affected us badly that we were stressed because of the situation.”* ***(FGD female out of school: Transcript 16)*** |
|  | - 1. Poor coping mechanisms | - | 2 | *“Some adolescents looked at the world ending so they lost hope, they developed more stress than before, they involved themselves in early marriages and more alcohol abuse while others developed those suicidal thoughts and some even lost their lives.”* ***(KII female teacher: Transcript 4)*** |
